# On Max-Plus Algebra and Its Application on Image Steganography

**DOI:** 10.1155/2018/6718653

**Published:** 2018-05-15

**Authors:** Kiswara Agung Santoso, Fatmawati, Herry Suprajitno

**Affiliations:** ^1^Department of Mathematics, Faculty of Mathematics and Natural Science, Jember University, Kampus Bumi Tegal Boto, Jl. Kalimantan 37, Jember 68121, Indonesia; ^2^Department of Mathematics, Faculty of Science and Technology, Universitas Airlangga, Kampus C, Jl. Mulyorejo, Surabaya 60115, Indonesia

## Abstract

We propose a new steganography method to hide an image into another image using matrix multiplication operations on max-plus algebra. This is especially interesting because the matrix used in encoding or information disguises generally has an inverse, whereas matrix multiplication operations in max-plus algebra do not have an inverse. The advantages of this method are the size of the image that can be hidden into the cover image, larger than the previous method. The proposed method has been tested on many secret images, and the results are satisfactory which have a high level of strength and a high level of security and can be used in various operating systems.

## 1. Introduction

Recently information systems are developing very quickly, especially information systems through the Internet. It happens because the Internet can be accessed by anyone, anytime, and anywhere. Access to information through the Internet does not always bring benefits but also risks to the accuracy of information. This risk is vulnerable when information is accessible by hackers.

Many efforts have been made to protect data transferred over the Internet, including encryption (protecting data before being transferred over the Internet) and authentication (verifying whether the received data is the same as the sent data). There is knowledge or art of data protecting transferred over the Internet, that is, cryptography (data encoding) and steganography (data disguise). The data to be discussed in this paper is image data.

Many steganography methods in protecting information into an image have been published. The data or information that is hidden into an image can be text data or image data. Generally, to hide text data or image data into another image, the original text and original image should be converted into binary digits (bits). Then each digit of the original image or original text is substituted into the last bit of the cover image pixel. By using this method, information could be hidden into the cover image with little difference between the image stego image and the cover image. In this algorithm, every character (for text) or pixel (for image) is hidden into three pixels of cover image. The consequence of this method is, for the text data, the maximum number of characters that can be hidden into the cover image is one-third of the total pixels of the cover image. For image data, the image size that can be hidden into the cover image is one-third the size of the cover image (either long or wide).

In modern world, all information communication is done online. It causes the security system when data transfer becomes very important. Steganography has its own mechanism in data protecting [1, 2]. In steganography, the information to be sent is hidden into other media, so that no one knows where the information is hidden. Watermarks and fingerprints are two technologies related to steganography, where steganography tends to hide data in other media [3].

Currently research on image encoding generally focuses on the following aspects: image encoding with spatial domains, image coding with domain transformation, image coding based on neural network, chaotic image coding, image coding based on cellular automata, and quantum technology [4]. In cryptography, encoding is the process of transforming information using certain algorithms that make it unreadable by anyone except the one who knows the special information, commonly called a key. The result of this process is called encrypted information [5]. Bouquard et al. have introduced the image encoding algorithm using affine transformation [6]. In this algorithm, the encryption and decryption process pass through two stages; that is, the first stage encodes the image using XOR operations with four key bits and the second stage encodes the encoded image using affine transformation. The conclusion of the study states that the correlation of pixel values between the original image and the encrypted image decreased after transforming the affine transform.

Tom has implemented data disguise using stenographic techniques. To make the technique safer they added a level of security by applying cryptography to confidential data before using steganography [7]. For cryptography, they use the Caesar algorithm while for steganography they use the adjacent pixel differences algorithm. Kulkarni and Jatgap substituted secret messages using a 14-square substitution algorithm [8]. Once the text was substituted, then this message was encoded with the RSA algorithm. The next step, this encoded message was hidden into an image by LSB (Least Significant Bit) method. This image works as a carrier file, which will be sent to the recipient. The receiver decrypts to get the original message by performing the same method but in reverse order. Here, it appears that they do two coding techniques, so the system becomes more powerful and secure in the face of hacker attacks. This technique makes it difficult for the troublemakers to manipulate the image and takes a long time to encrypt the message, so it is safe from various attacks through the Internet network.

In measuring quality of an image objectively, some data are statistically calculated to determine quality of the reconstruction image. Image quality could be seen from how close the relationship of image forming pixels or by looking at how much the difference in pixel values are statistically distributed. In general, to compare two images, one could use mean square error (MSE) and Peak to Signal Noise Ratio (PSNR) [9, 10]. Choudhary applied the optimization process to a stego image by using the LSB method, so that quality of the stego can get better with lower computational complexity [11]. MSE between stego image and cover image can be derived. Experimental results show that visually the stego image cannot be distinguished from the cover image. The results also showed improvement compared to the previous one.

In this paper, we propose a new steganography method to hide an image into another image using matrix multiplication operations on max-plus algebra. This is especially interesting because the matrix used in encoding or information disguises generally has an inverse, whereas matrix multiplication operations in max-plus algebra do not have an inverse. Another advantage of this method is the size of the image that can be hidden into the cover image which is greater than using the previous method.

## 2. Max-Plus Algebra

Max-plus algebra can be used to model disk events related to synchronization and time delays. The application of this theory has a very strong association with production problems [12, 13].

The max-plus algebra [7] is a sequential pair (*R*, ⊕, ⊗), where *R* is the set of all real numbers, whereas ⊕ and ⊗ are binary operations on *R* defined as

(1) a⊕b=max⁡a,b,a⊗b=a+b

for every *a*, *b* ∈ *R*. Operations ⊕ and ⊗ are extensions of matrices and vectors in the same way as conventional linear algebra.

In the max-plus algebra [6], the matrix multiplier *A* ⊗ *B* is defined as follows: for any matrix *A* ∈ *R*^*m*×*p*^, *B* ∈ *R*^*p*×*n*^, we can obtain matrix *C* ∈ *R*^*m*×*n*^ by the formula

(2) $$\begin{array}{c} c_{ij}=\overset{p}{\underset{k=1}{⨁}}\left(\;a_{ik}⊗b_{kj}\;\right) \end{array}$$

for *i* = 1,…, *m*, *j* = 1,…, *n*. For a square matrix with degree *k*, matrix *A* ∈ *R*^*n*×*n*^ denoted by *A*(*k*) and was defined by recursive operation on *k* = 2,3,…:

(3) $$\begin{array}{c} A\left(\;k\;\right)=A⊗A^{\left(k-1\right)}. \end{array}$$

The set of *R*_max_ with operations ⊕ and ⊗ is called max-plus algebra and denoted by *R*_max_ = (*R*_max_, ⊕, ⊗, *ε*, *e*). As conventional algebra, operations ⊗ have a higher priority than ⊕. For example, operation 5 ⊗ −9 ⊕ 7 ⊗ 1 has an understanding like (5 ⊗ −9)⊕(7 ⊗ 1).

Note that (5 ⊗ −9)⊕(7 ⊗ 1) = 8, where 5 ⊗ (−9 ⊕ 7) ⊗ 1 = 13.

In addition, there is −∞ such that max⁡(*a*, −*∞*) = max⁡(−*∞*, *a*) = *a* and *a* + (−*∞*) = −*∞* + *a* = −*∞*. For any *a* ∈ *R*_max_, there is a small number *ε* such that

(4) a⊕ε=ε⊕a=a,a⊗ε=ε⊗a=ε.

Let *A* ∈ *R*_*ε*_^*n*×*n*^ and *b* ∈ *R*_*ε*_^*n*^. In general, the system of linear equations in max-plus algebra will have no solution, if *A* is square matrix or if the number of columns in *A* is more than the number of rows in *A*. Therefore, subsolutions concepts are introduced [7].

Operator ⊗ is a commutative operator. Except 0, every element has an inverse. The inverse of *x* is denoted by *x*^−1^ or 1/*x*. More precisely, we denote *x*/*y* or *x* ⊗ *y*^−1^. *x* ⊗ *y* multiplication could be denoted by *xy*. The operator allows it to be expanded to a *m* × *m* matrix on *R*_max_ [14].

Let *A* and *B* be two matrices of *m* × *m*, operator ⊕, and we define

(5) A⊕Bi,j=Ai,j⊕Bi,j,∀i,j∈1,…,m2,A⊗Bi,j=⨁k=1mAi,k⊗Bk,j,∀i,j∈1,…,m2.

It is not difficult to prove that the *m* × *m* matrix exists in *R*_max_. Based on the triangular matrix *A* of size *m* × *m*, where *A*_*i*,*j*_ = 0 for *i* > *j*, it is indicated that the set of *m* × *m* triangular matrices exists in *R*_max_, but the operator ⊗ is not commutative. Furthermore, not all elements in max-plus algebra have inverse [6].

## 3. Literature Review

The image data character is very different from the text data because an image contains very large data, and all data has a very strong relationship and contains very high data loops [15].

Conceptually, the difference between text data and image data can be seen in Table 1.

An image is defined as a two-dimensional function, *f*(*x*, *y*), where *x* and *y* are spatial coordinates and *f* is the light intensity at coordinates (*x*, *y*) known as the gray degree. An image is called a digital image if, in position (*x*, *y*), there is an amplitude value. A digital image constitutes a finite number of elements, each of which has a particular location and a particular value. These elements are called picture elements or images of elements or pixels [16].

**Figure 1:**
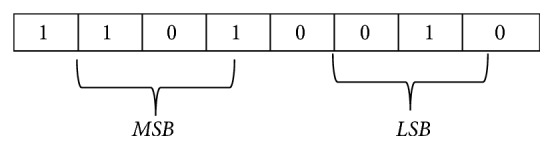
MSB and LSB bit.

The pixel of an image can be converted into 8 binary digits (bits). The first to fourth bit is called LSB (Least Significant Bit) where the bit value changes in this position have no impact on the image. The fifth to eighth bit is called MSB (Most Significant Bit), where changes in bit values in this position have an effect on image.  Figure 1 shows the bit positions of MSB and LSB.

The maximum deviation of an image can be found by making a grayscale histogram and calculating its area. The larger the deviation is, the better the encoding will be. To find the area of deviation image can be seen from the following formula [17]:

(6) $$\begin{array}{c} L=\frac{h_{0}+h_{255}}{2}+\overset{254}{\underset{i=1}{\sum }}h_{i}. \end{array}$$

Here,

 
*L* is the area of deviation;
 
*h*_*i*_ is the number of pixels that have gray degree;
 
*i* is the value of pixels.

A simple example of hiding data into an image is called insertion of least significant bit (LSB). For 24-bit colored images, the number of changes will be minimized so that it is difficult to distinguish by the human eye. For example, suppose we have three adjacent pixels (nine bytes) by using RGB encoding. Suppose we will hide data 101101101. Put 9 bits of data in the LSB position, so the following pixels are obtained (bold font shows the changed bits):

(7) $$\begin{array}{c} \begin{array}{ccc} 10010101 & 00001101 & 11001001\\ 10010110 & 00001111 & 11001010\\ 10011111 & 00010000 & 11001011 \end{array} \end{array}$$

**Figure 2:**
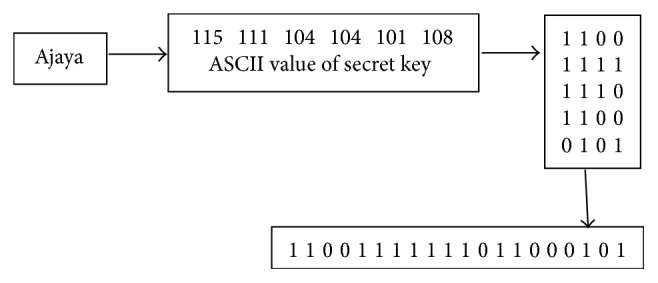
1D array representation.

Based on the formula, here is a snippet of the steganography process (see Figure 2).

*Application of stenographic LSB uses secret key*. Kulkarni and Jatgap [8] take a binary representation to hide information and replace the LSB of each cover image bit. Here, a secret key is introduced to protect the hidden information by using the formula:

(8) Cover  image+secret  key+hidden  information=stego  image.

To hide information, one should use cover image. Cover image is divided into three matrices (Red, Green, and Blue). Secret key is converted to 1D bit stream array. Secret key and Red matrix are used as decision-makers to replace hidden information into the Green matrix or Blue matrix. Every bit of the secret key is operated by operators XOR with every LSB bit on the Red matrix. The result of the XOR operation is used to determine the bit of the hidden information to be replaced in the Green matrix LSB or the Blue matrix. The same process is done until all information is successfully hidden [18]. In this method, every character (plain text) or every pixel (plain image) is hidden into three pixels of cover image. As a consequence of this method, for plain text, the maximum number of characters that can be hidden into the cover image is 1/3 of the total pixel cover image. The maximum size of plain image that can be hidden is 1/3 of the size of the cover image (for length and width).

In the previous method, it is required that the size of the secret image should be smaller than the size of the cover image. In this article, we propose a new method so that the size of the secret image can be increased to the same size as the cover image.

## 4. The Proposed Method

The following algorithm is how to hide secret image into another image with maximal size equal to cover image size:

1. Convert pixels from secret image and cover image into bitwise form.
2. Change the MSB from the pixel cover to the 2 × 2 matrix form.
3. Find the secret image matrix using
  - *SR*_(*i*,*j*)_ = *R*_(*i*,*j*)_ ⊗ *G*_(*i*,*j*)_,
  - *SG*_(*i*,*j*)_ = *G*_(*i*,*j*)_ ⊗ *B*_(*i*,*j*)_,
  - *SB*_(*i*, *j*)_ = *B*_(*i*, *j*)_ ⊗ *R*_(*i*,*j*)_,
  - where
     
*R*_(*i*,*j*)_ is the MSB of cover image matrix on the Red layer at position (*i*, *j*),
     
*G*_(*i*,*j*)_ is the MSB of cover image matrix on the Green layer at position (*i*, *j*),
     
*B*_(*i*,*j*)_ is the MSB of cover image matrix on the Blue layer at position (*i*, *j*).
4. Substitute the MSB secret image into the LSB stego image with the following rules:
  - If *a*_11_ > *a*_12_, then substitute the first bit of MSB_si_ into the second bit of LSB_ci_ and substitute the second bit of MSB_si_ to the first bit of LSB_ci_.
  - If *a*_11_ ≤ *a*_12_, then substitute the first bit of MSB_si_ into the first bit of LSBci and substitute the second bit of MSB_si_ into the second bit LSB_ci_.
  - If *a*_21_ > *a*_22_, then substitute the third bit of MSB_si_ into the fourth bit of LSB_ci_ and substitute the fourth bit of MSB_si_ into the third bit of LSB_ci_.
  - If *a*_21_ < *a*_22_, then substitute the third bit of MSB_si_ into the third bit of LSB_ci_ and substitute the fourth bit of MSB_si_ into the fourth bit of LSB_ci_.

Here

 
*a*_*ij*_ is the element of matrix of the secret image at row *I* and column *j*.
 
MSB_sc_ is the MSB of secret image.
 
LSB_st_ is the LSB of stego image.

**Figure 3:**
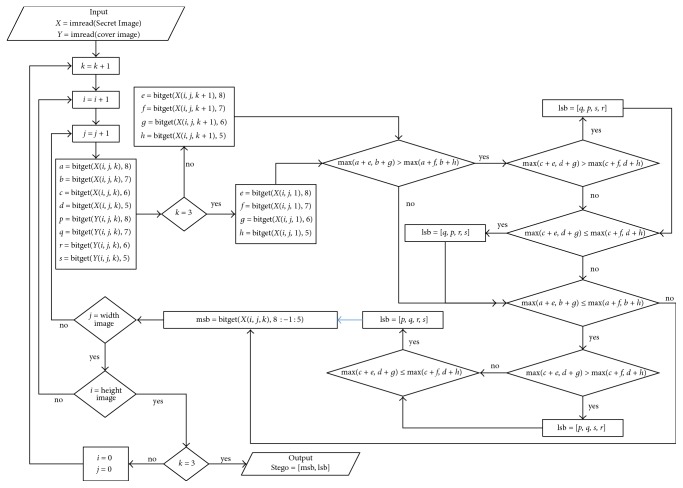
Flowcharts to hide the secret image into the cover image.

For more details, the proposed coding system can be illustrated through the flowchart as in Figure 3.

Here is an algorithm to display the secret image of the stego image.

1. Convert pixels of stego image into 8-bit form.
2. Calculate the secret image matrix by
  - *SR*_(*i*,*j*)_ = *R*_(*i*,*j*)_ ⊗ *G*_(*i*,*j*)_,
  - *SG*_(*i*,*j*)_ = *G*_(*i*,*j*)_ ⊗ *B*_(*i*,*j*)_,
  - *SB*_(*i*,*j*)_ = *B*_(*i*,*j*)_ ⊗ *R*_(*i*,*j*)_.
  - Here
     
*R*_(*i*,*j*)_ is the LSB of stego image matrix on the Red layer at position (*i*, *j*).
     
*G*_(*i*,*j*)_ is the LSB of stego image matrix on Green layer at position (*i*, *j*).
     
*B*_(*i*,*j*)_ is the LSB of stego image matrix on the Blue layer at position (*i*, *j*).
3. Exchange LSB bitwise LSB of stego image with the following rules:
  - If *a*_11_ > *a*_12_, then exchange the first bit with the second bit.
  - If *a*_21_ > *a*_22_, then exchange the third bit with the fourth bit.
  - Here *a*_*ij*_ is the matrix element of the secret image matrix on the row *I* and column *j*.
  - Convert LSB and MSB from the stego image.
  - Exchange bit 1 with bit 5.
  - Exchange bit 2 with bit 6.
  - Exchange bit 3 with bit 7.
  - Exchange bit 4 with bit 8.

**Figure 4:**
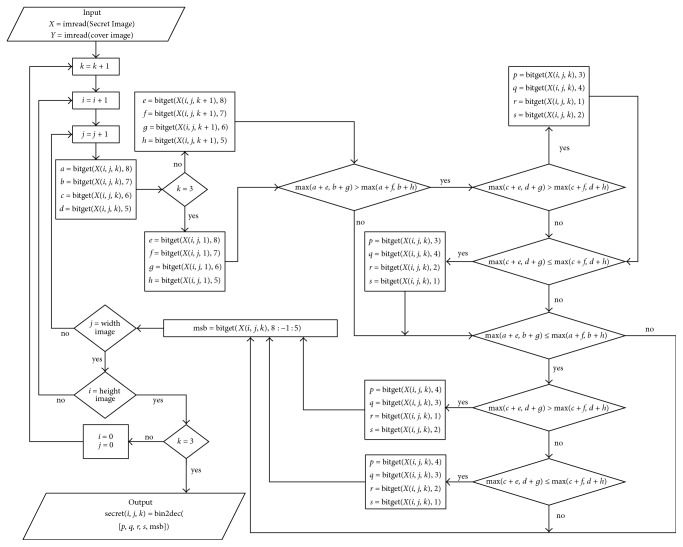
Flowchart to display the secret image of the stego image.

For more details, decryption algorithm that can be made through the flowchart as in Figure 4.

In Table 2, the example pixel data from the secret image and the cover image is presented.

We process the following operations: *SR* = *R* ⊗ *G*  *SG* = *G* ⊗ *B*  *SB* = *B* ⊗ *R*:

(9) SR=0001⊗0110=1121,SG=0110⊗1001=1221,SB=1001⊗1001=2112.

In the matrix column in Table 3, if element of the left matrix is larger than element of the right matrix, then the element will be exchanged. The results of the operation process in Table 3 are given in Table 4.

## 5. Experimental Results and Analysis 

**Figure 5:**
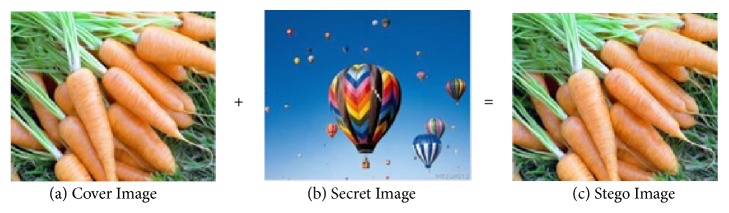
Experimental result.

To test our method, an experiment was performed. Here the test is done by using a laptop with microprocessor core i3 and Microsoft Windows 10 operating system. Computer program was created by using MATLAB R2016b and it applied to an image of good quality. The results of our algorithm are shown in Figure 5. We use the balloon image as a secret image and carrot image as the cover image. Cover image and secret image have the same size that is 163 × 133.

Figure 5 shows that the stego image (Figure 5(c)) is similar to the cover image (Figure 5(a)), although visually inside the cover image contains a secret image (Figure 5(b)). The result of stego image has a size of 163 × 133. It is proved that this method can hide the secret image that has size as same as the cover image. This needs to be demonstrated by using statistical analysis. Therefore, an ideal encoding must have power when there is an attack through its statistical model. To prove the power of this proposed method, we performed a statistical analysis by displaying a histogram and computing the correlation coefficient between two neighboring pixels on the cover image and stego image.

**Figure 6:**
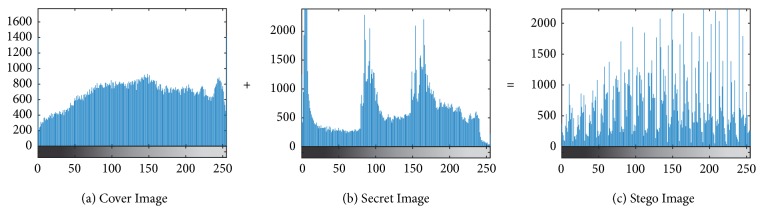
Histogram analysis.

The abscissa histogram shows the pixel value and the ordinate showing the frequency or how often the pixel value appears. The histogram of the cover image shown in Figure 6(a) has a larger area. This area shows how often the pixel value appears in an image. Histogram of the secret image shown in Figure 6(b) has a smaller area. This shows that the cover image is clearer than the secret image. Histogram of the stego image shown in Figure 6(c) has a pattern similar to the cover image. This shows that the cover image has not significantly changed.

In addition to histogram analysis, we also analyze the correlation coefficients of two vertically neighboring pixels, two horizontally neighboring pixels, and two pixels diagonally adjacent to the stego image and cover image. First, we select 10000 pixels on a neighboring image. Then we calculate the correlation coefficient with the following formula:

(10) cov⁡x,y=Ex−Exy−Ey,rxy=cov⁡x,yDxDy.

Here, *x* and *y* are the values of two neighboring pixels. In numerical computation, the correlation coefficient can be calculated using the following formula [19]:

(11) Ex=1N∑i=1Nxi,Dx=1N∑i=1Nxi−Ex2,covx,y=1N∑i=1Nxi−Exy−Eyi.

Based on the proposed method, the correlation coefficient between two vertically neighboring pixels for the cover image and stego image is 0.91432 and 0.91039, respectively. Similarity of the results to the vertical and diagonal directions is shown in Table 5. It is apparent in Table 5 that there is a strong correlation between two neighboring pixels, or in other words the stego image and cover image are difficult to distinguish.

In the image processing mean square error (MSE) is often used to determine how big the image quality difference between before and after coding process. The formula is presented as follows [15]:

(12) $$\begin{array}{c} {MSE}_{image}=\frac{1}{MN}\overset{M}{\underset{y=1}{\sum }}\;\overset{N}{\underset{x=1}{\sum }}{\left|\;I\left(\;x,y\;\right)-I^{\prime }\left(\;x,y\;\right)\;\right|}^{2}, \end{array}$$

where

 
*M* is the length of image (in pixel),
 
*N* is the width of image (in pixel),
 
*I*(*x*, *y*) is the initial image pixel value,
 
*I*′(*x*, *y*) is the resulting image pixel value.

Based on the calculation, the MSE between the cover image and stego image is 361.7734

Improving the visual quality of digital image can be subjective. Saying that one method provides better quality image could vary from person to person. Using same tests images, different image enhancement algorithm can be compared by peak signal to noise ratio (PSNR). The mathematical representation of the PSNR is as follows:

(13) $$\begin{array}{c} PSNR=20{\;\log}_{10}\left(\;\frac{{MAX}_{f}}{\sqrt{MSE}}\;\right), \end{array}$$

where MAX_*f*_ is the maximum signal value that exists in our original “known to be good” image. Two identical images will have a zero MSE value and an infinite PSNR value so the smaller the difference between the two images, the smaller the MSE value and the larger the PSNR value [19].

From the calculation results obtained PSNR for cover image is 21.8295 and PSNR for stego image is 21.8142. Here it looks very small value difference so it can be said that the image between cover image and stego image is similar. With this similar result it can be said that the coding result goes well.

Based on analysis of time, this algorithm has the time complexity *O*(*n*). This shows that the algorithm has execution time that increases linearly according to the number of pixels (image size).

*Comparison of Max-Plus Methods and Previous Methods.* To compare the proposed coding method with the previous coding method, we use koala image (1024 × 768) as a secure image and tulips image (1024 × 768) as cover image.

**Figure 7:**
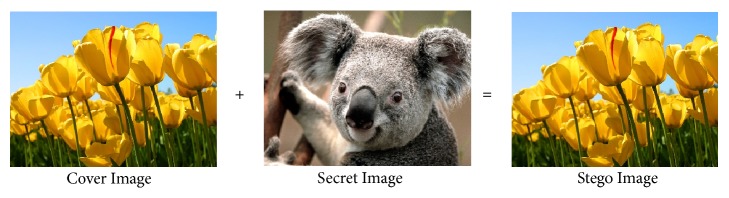
Encoding by max-plus algebra.

**Figure 8:**
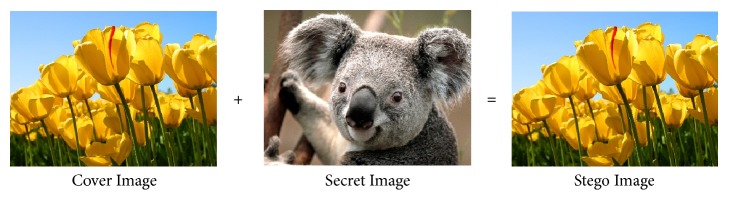
Encoding by previous method.

**Figure 9:**
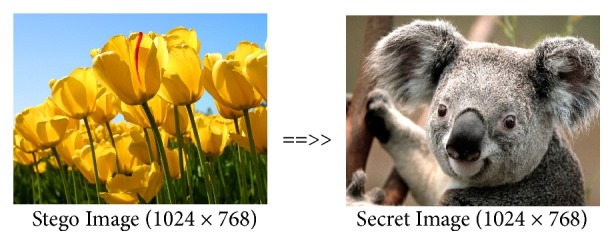
Decoding by max-plus algebra.

**Figure 10:**
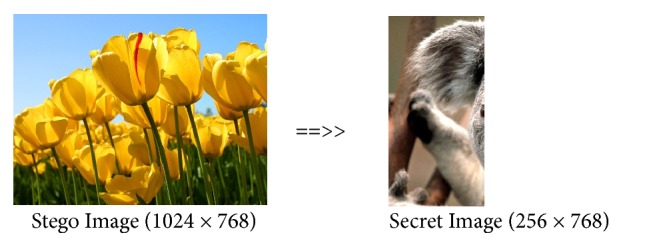
Decoding by previous method.

From Figures 7 and 8, we can see that the encoding between the max-plus and the previous method produces the same stego image. Both methods can be used to hide the secret image that has same image size between the secret image and the cover image. Figures 9 and 10 show the results of decoding by max-plus and previous methods. Hence, we conclude that the decoding process of the Max-Plus method can return the stego image same as the secret image, while in the previous method it cannot return the stego image to the secret image perfectly but only a quarter of the part. It makes the previous method display a quarter of the secret image during the description process.

## 6. Conclusions

The proposed method has been tested on many secret images, and the results are satisfactory which have a high level of strength and a high level of security and it can be used in various operating systems. A pixel of the secret image is hidden in a cover image pixel by matrix multiplication operations in max-plus algebra, so that the message becomes safer. Maximum secret image size that can be hidden is the same as the size of cover image. This is the novelty of this method where, in the previous method, size of secret image is always smaller than the cover image. In our future research, we will construct an algorithm to hide a text into an image by using max-plus algebra.

## Figures and Tables

**Table 1:** The difference between text encoding and image encoding.

Type	Secret data	Encrypt data	Remarks
Text	CSEMCKVIE	DTENDLWJF	Different

Image (RGB)	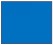	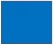	*P*_1_ and *P*_2_ are different, but visually they are difficult to distinguish
	Pixel(*P*_1_) = (24,45,233)	Pixel(*P*_2_) = (10,65,198)	

Image (Gray)	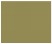	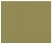	*P*_1_ and *P*_2_ are different, but visually they are difficult to distinguish
	Pixel(*P*_1_) = (87)	Pixel(*P*_2_) = (114)	

**Table 2:** Example data.

Image	Layer	Value	Bit	MSB	LSB
Secret image	Red	79	00010001	0001	0001
	Green	108	01101100	0110	1100
	Blue	205	11001101	1100	1101

Cover image	Red	28	00011100	0001	1100
	Green	104	01101000	0110	1000
	Blue	146	10010010	1001	0010

**Table 3:** Operation process.

Secret image		Matrix	Stego image	
MSB	Process		Process	LSB
0001	00 01	*SR*: $\left(\begin{array}{c} a_{11}=a_{12}\\ a_{21}>a_{22} \end{array}\right)$	00 10	0010
0110	01 10	*SG*: $\left(\begin{array}{c} a_{11}<a_{12}\\ a_{21}>a_{22} \end{array}\right)$	01 01	0101
1100	11 00	*SB*: $\left(\begin{array}{c} a_{11}=a_{12}\\ a_{21}<a_{22} \end{array}\right)$	11 00	1100

**Table 4:** The results.

Image	Layer	Value	Bit	MSB	LSB	Matrix
Cover	*R*	28	00011100	0001	1100	$R=\left(\begin{array}{cc} 1 & 0\\ 0 & 1 \end{array}\right)$
	*G*	104	01101000	0110	1000	$G=\left(\begin{array}{cc} 0 & 1\\ 1 & 0 \end{array}\right)$
	*B*	146	10010010	1001	0010	$B=\left(\begin{array}{cc} 1 & 0\\ 0 & 1 \end{array}\right)$

Secret	*R*	79	00010001	0001	0001	*SR* = $\left(\begin{array}{cc} 1 & 1\\ 2 & 1 \end{array}\right)$
	*G*	108	01101100	0110	1100	*SG* = $\left(\begin{array}{cc} 1 & 2\\ 2 & 1 \end{array}\right)$
	*B*	205	11001101	1100	1101	*SB* = $\left(\begin{array}{cc} 2 & 1\\ 1 & 2 \end{array}\right)$

Stego	*R*	18	00010010	0001	0010	
	*G*	101	01100101	0110	0101	
	*B*	156	10011100	1001	1100	

**Table 5:** The correlation coefficient.

Image	Horizontal	Vertical	Diagonal
Cover	0.97028	0.91432	0.97337
Stego	0.96014	0.91039	0.92923

## References

[1] Khurana A., Mehta B. (2012). Comparison of LSB and MSB based Image Steganography.

[2] Sharma A., Sharma V. (2015). Improved performance of secure data hiding algorithm using non blind steganography technique.

[3] Singh S., Sharma S. (2014). Data Hiding using difference between adjacent pixels and bit plane swapping.

[4] Kori P., Dubey P., Richhariya V. (2015). Double phase image encryption and decryption using logistic tent map and chaotic logistic map.

[5] Kester Q. (2013). Image Encryption based on the RGB PIXEL Transposition and Shuffling.

[6] Bouquard J.-L., Lenté C., Billaut J.-C. (2006). Application of an optimization problem in max-plus algebra to scheduling problems.

[7] Tom H. (2003). Max-plus algebra and its application in spreading of information Circulant matrices.

[8] Kulkarni M., Jatgap P. (2015). An efficient data hiding scheme using steganography and cryptography technique.

[9] Chandranath A. Robust steganography using LSB-XOR and image sharing.

[10] Kaur J. A Secure Technique for hiding data under the Fingerprint Images using Modified Haar Wavelet Based Transformation.

[11] Choudhary K. (2012). Image steganography and global terorism.

[12] Lini A., Neenu D. (2013). Secure image encryption algorithms: A review.

[13] Menguy E., Boimond J.-L., Hardouin L., Ferrier J.-L. (2000). Just-in-time control of timed event graphs: update of reference input, presence of uncontrollable input.

[14] Case B. (2010). Max-Plus Algebra : From Discrete-event Systems to Continuous Optimal Control Problems.

[15] Liu Z., Guo C., Tan J. (2015). Securing color image by using phase-only encoding in Fresnel domains.

[16] De Schutter B., Boom T. V. D. Max-plus algebra and max-plus linear discrete event systems: An introduction.

[17] Shin C.-M., Seo D.-H., Kim S.-J. (2004). Gray-level image encryption scheme using full phase encryption and phase-encoded exclusive-OR operations.

[18] Gangwar A. (2013). Improved RGB-LSB seganography using secret key.

[19] Liu Z., Chen H., Blondel W., Shen Z., Liu S. (2018). Image security based on iterative random phase encoding in expanded fractional Fourier transform domains.

